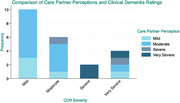# Discrepancies in Dementia Severity Perceptions: Exploring the Agreement Between Care Partners and Clinical Assessments

**DOI:** 10.1002/alz70858_106983

**Published:** 2025-12-26

**Authors:** Paige DiStefano, Harmonie Chan, Jyothy Nair, Malene Stewart, Samantha Shune, Nan Uzbalis, Ashwini Namasivayam‐MacDonald

**Affiliations:** ^1^ McMaster University, Hamilton, ON, Canada; ^2^ University of Oregon, Eugene, OR, USA

## Abstract

**Background:**

Informal family care partners are crucial in dementia care, serving as primary advocates and caregivers. A caregivers’ perception of their care recipient's dementia severity, whether they overestimate or underestimate the progression, can impact care satisfaction and applicability. Overestimation may lead to unnecessary interventions or distress, while underestimation can delay critical support. Caregivers offer valuable insights extending beyond clinical scores, capturing nuances essential for effective care planning. This study investigates the agreement between caregivers’ perception of dementia severity and clinical severity assessment, and how demographic and contextual factors influence accuracy.

**Method:**

Data were collected from a convenience sample of 22 dyads. Caregivers were eligible if they were 18 years or older, spoke and read English, and provided unpaid care to a family member with dementia for at least 2 months. Caregivers completed surveys ranking their perception of their care recipient's dementia severity (e.g., mild, moderate, severe, very severe), along with various demographic and contextual factors. A collapsed version of the Clinical Dementia Rating (CDR) scale, administered by certified staff, objectively measured dementia severity. Agreement between caregivers’ perceived dementia severity and CDR severity was evaluated, and additional analyses identified predictors of accuracy.

**Result:**

Forty‐five percent of caregivers (age = 62.8±14.7; 82% female) overestimated their care recipients’ (age = 81±9.6; 50% female) dementia severity (i.e., 70% of mild cases; 17% of moderate; 100% of severe), and eighteen percent underestimated severity (i.e., 17% of moderate cases; 75% of very severe). There was a significant positive association between caregivers’ perceived dementia severity and CDR severity (*p* = 0.041), indicating that perceived severity increased as CDR severity increased (OR=2.4). There was weak agreement between perceived and CDR severity (K = 0.1; *p* >0.05). No demographic or contextual factors influenced perception accuracy.

**Conclusion:**

While caregivers’ perceptions of dementia severity often diverge from clinical assessments, they reflect the lived reality. Acknowledging the tendency to overestimate, improved communication and education between dyads and providers is vital. Dynamic knowledge sharing will help align expectations and ensure sufficient support is provided based on the needs of the care recipient, not solely their clinical score.